# Lifestyle INtervention for Diabetes prevention After pregnancy (LINDA-Brasil): study protocol for a multicenter randomized controlled trial

**DOI:** 10.1186/s12884-016-0851-x

**Published:** 2016-03-30

**Authors:** Maria Inês Schmidt, Bruce B. Duncan, Cristina Castilhos, Eliana Márcia Wendland, Pedro C. Hallal, Beatriz D’Agord Schaan, Michele Drehmer, Adriana Costa e Forti, Cristina Façanha, Maria Angélica Nunes

**Affiliations:** Postgraduate Program in Epidemiology, School of Medicine, Federal University of Rio Grande do Sul, R. Ramiro Barcelos, 2600, Sala 414, Porto Alegre, RS 90035-003 Brazil; Hospital de Clínicas de Porto Alegre, Porto Alegre, RS Brazil; Department of Collective Health, Federal University of Health Sciences, Porto Alegre, RS Brazil; Postgraduate Program in Epidemiology, Federal University of Pelotas, Pelotas, RS Brazil; Postgraduate Studies Program in Endocrinology, School of Medicine, Federal University of Rio Grande do Sul, Porto Alegre, RS Brazil; Department of Internal Medicine, School of Medicine, Federal University of Ceará, Fortaleza, Ceará Brazil; Integrated Center for Diabetes and Hypertension, Ceará State Health Department, Fortaleza, Ceará Brazil; School of Medicine, Centro Universitário Unichristus, Fortaleza, Ceará Brazil

**Keywords:** Gestational diabetes, Type 2 diabetes mellitus, Randomized controlled trial, Telemedicine, Weight loss

## Abstract

**Background:**

Gestational diabetes mellitus (GDM), a hyperglycemic state detected during pregnancy, is an established risk factor for diabetes. However, treatment during pregnancy in and of itself is not able to eliminate this risk, and a considerable fraction of women with GDM will develop frank diabetes in the decade following pregnancy. Our aim is to conduct a multicenter randomized controlled trial to investigate the effectiveness of a lifestyle intervention program implemented after a pregnancy complicated by GDM in delaying or preventing the development of type 2 diabetes.

**Methods:**

Women aged 18 or older identified as having recent GDM are recruited and followed by telephone to assess eligibility for the trial. To be eligible, women must have used insulin during pregnancy or present intermediate hyperglycemia postpartum. Women are encouraged to enter the trial as early as 10 weeks, and are permitted to do so up to 2 years after a pregnancy with GDM. An estimated 740 women will be randomized to either conventional care or to coach-based interventions focused on breastfeeding, weight loss, healthy eating, and increased physical activity, and predominantly delivered by telephone. Women are followed annually to detect new onset diabetes, the primary outcome, and additional secondary outcomes which include reversion to normoglycemia, weight loss, physical activity and fitness, and insulin resistance.

**Discussion:**

Though previous studies have demonstrated that type 2 diabetes can be delayed or prevented, no study has yet demonstrated the feasibility and effectiveness of similar interventions implemented in the postpartum period for women with recent GDM. If shown to be successful, this approach could become an important means of preventing diabetes in primary care settings.

**Trial registration:**

ClinicalTrials.gov Identifier: NCT02327286; Registered 23 December 2014.

## Background

Diabetes is a major cause of morbidity and mortality and is one of the four main chronic diseases identified by the World Health Organization as the focus for prevention and control [1]. Diabetes can be prevented, as shown by two landmark clinical trials, the Diabetes Prevention Program (DPP) and the Diabetes Prevention Study (DPS): an average of three years of lifestyle interventions can reduce by 58 % the incidence of type 2 diabetes in individuals presenting impaired glucose tolerance [2, 3]. Subgroup analyses of one of these trials, focusing on women with previous gestational diabetes mellitus (GDM), found a 53 % reduction at the end of the trial [4] and a 35 % reduction 10 years later [5].

GDM, a hyperglycemic state detected during pregnancy, is an established risk factor for diabetes [6] but treatment during pregnancy in and of itself is not able to eliminate its risk [7]. Although a clinical trial showed that troglitazone could reduce diabetes incidence by 55 % when applied to very high risk women with GDM, the drug was subsequently withdrawn from the market due to serious adverse effects [8].

More recent randomized trials are testing the effect of lifestyle interventions after pregnancy among women with GDM with the aim of preventing diabetes. One such trial randomized 450 women with GDM and impaired glucose tolerance postpartum in China and found that the incidence of diabetes did not differ between the intervention (15 %) and the control group (19 %), after 36 months of follow up [9]. Four other trials are ongoing. One, in China, is randomizing 1180 women with GDM diagnosed over a prior 5-year period [10]. Another, in Australia, is randomizing women at postpartum with the aim of reducing diabetes risk at 12 months postpartum [11]. A third is a pragmatic cluster randomized clinical trial of 44 medical facilities at Kaiser Permanente Northern California including 2320 women with GDM, having as primary outcomes postpartum weight goals at 6 and 12 months [12]. Finally, an additional randomized trial is testing the efficacy of an individually-tailored lifestyle intervention to reduce risk factors for type 2 diabetes and cardiovascular disease among postpartum Hispanic women with a history of abnormal glucose tolerance during pregnancy [13].

### Rationale for the trial and for the choice of the interventions

Given the increased burden of diabetes worldwide, preventive actions are urgently needed. Lifestyle modifications have been shown to be effective in the prevention of diabetes when offered to high risk middle-age individuals, but identification of those at risk is challenging. Pregnancy is a good opportunity to identify women at risk since glucose testing is routinely done for the detection of gestational diabetes. Since retesting at postpartum is also routinely recommended for these women, those found to be at higher risk can be targeted for early diabetes prevention. Moreover, lifestyle interventions are usually implemented during pregnancy for the treatment of gestational diabetes. Since women frequently abandon these lifestyle changes after pregnancy, interventions to encourage a healthy lifestyle are needed and must be tailored to the postpartum period so as to take into account the difficulties involved in this setting of adapting to the needs of the new baby.

Motivational interviewing [14] and other communication strategies [15, 16] may help tailor the intervention to women’s health needs and stimulate behavioral change in the context of coping with the demands of motherhood. The choice of the interventions must be focused on the potential benefits for these women at this particular phase of their lives. In this regard, a few interventions are especially suitable, as discussed below.

Breastfeeding, a healthy behavior in and of itself, has been associated with improved maternal metabolic profiles when assessed by observational studies [17]. Breastfeeding was associated with lower blood glucose levels 6–12 weeks postpartum in women with GDM [18], greater postpartum weight loss [19], lower risk of obesity in the long run, as well as lower risk for the metabolic syndrome [20, 21]. Breastfeeding exclusively for 6 months, and to any extent for 12 months, reduced weight retention at 6 months postpartum, irrespective of pre-pregnancy body mass index (BMI); and at 18 months postpartum in women with BMI < 35 kg/m^2^ [22, 23]. Moreover, a decreased risk of diabetes has been found for breastfeeding women with and without GDM, although not in all studies [22, 24–27]. Despite these potential benefits, obese women have shorter lengths of exclusive and overall breastfeeding [28, 29], and those obese and also with diabetes or GDM, lower rates of intending and initiating breastfeeding [30].

Weight control interventions to facilitate achieving pre-pregnancy weight and further weight loss at postpartum are fundamental for the prevention of diabetes, as major diabetes prevention trials have shown that a loss of about 5 % of body weight is associated with a decreased incidence of diabetes [2, 3]. Pre-pregnancy weight [31], pregnancy weight gain and retention of weight at postpartum [7] are common and important risk factors for type 2 diabetes in GDM women. A recent Brazilian pregnancy cohort reported excessive weight gain in 44.8 % of women, being more frequent among those who were overweight prior to pregnancy [32]. The same study found median weight retention at 4–6 months postpartum of 4.4 kg, with 33 % of women moving to a higher BMI category and an additional 15 % of women who were already overweight gaining additional weight without a change of BMI category.

Evidence summarized by a Subcommittee on Nutrition During Lactation (Institute of Medicine) shows that lactating women typically lose weight at the rate of 0.5 to 1.0 kg per month in the first 4 to 6 months of lactation, probably related to physiologic changes during this period. The Committee also noted that some women lose as much as 2 kg per month and successfully maintain milk volume. The Subcommittee recommended that during lactation daily energy intake should not be restricted to less than 1800 kcal and especially not to less than 1500 kcal [28].

Further studies have demonstrated that to promote a weight loss of 2 kg/month (0.5 kg/week), overweight lactating women may restrict their usual energy intake by 500 kcal/day and exercise aerobically 4 days/week [33]. A Cochrane systematic review supports the feasibility and safety of moderate dietary restrictions (alone or in combination with exercise) for postpartum weight loss [34]. A further systematic review demonstrated that exercise strategies in postpartum women, with or without dietary intervention, improve weight loss compared with usual care [35]. A recent trial based on 2.5 h of individual sessions and biweekly phone interaction to stimulate weight loss through healthy diet and physical activity found that a 12-weeks intervention in lactating women (overweight or obese before pregnancy) produced an average weight loss of 8.3 kg shortly after the intervention and of 10.2 kg at 1-y follow-up [36], with improvement of cardiovascular risk factors [37]. However, given the high level of education of these women, it is important to consider also the more modest effects observed in a pilot study of lifestyle interventions for women with GDM during pregnancy and the postpartum period, oriented mostly by phone, which was conducted in a less favorable socioeconomic context [38].

The DPP and the DPS [2, 3], focused their dietary interventions on a decrease in calories and fats, especially saturated fats, and the DPS also on an increase in fiber. However, it is also important to consider new knowledge that has developed since the publication of these studies. An intervention based on a Mediterranean diet, supplemented with either olive oil or nuts, was more effective than a low-fat diet in the prevention of diabetes [39]. Systematic reviews of observational studies suggest that increased consumption of red meat and especially processed meat are related to a higher incidence of diabetes [40], and that increased consumption of dairy products is related to a lower incidence [41, 42]. Additionally, a systematic review of observational studies has strengthened the evidence that increased consumption of food rich in fiber is associated to a lower incidence of diabetes [43]. Consumption of ultra-processed foods, likely to play an important role in the current obesity epidemic, may also be related to the incidence of diabetes. In Brazil, sugars, margarine, salt and pasta already seasoned for preparation, as well as ultra-processed foods (fast foods and convenience foods such as snacks and desserts which are pre-prepared and ready-to-eat) are increasingly being consumed [44], and ultra-processed foods have been found to be associated with obesity [44, 45].

Artificial sweeteners, frequently used in Brazil to sweeten coffee and tea, have been recently shown to drive the development of glucose intolerance in rodents through direct compositional and functional alterations in the intestinal microbiota [46]. In humans the association between artificially sweetened beverage intake and glycemic level is inconsistent, and positive associations have been suggested to be due to reverse causality [47]. Coffee (independent of being decaffeinated or not) as well as tea consumption have been related to lower risk for diabetes in meta-analyses of observational studies [48]. Although both are a major source of anti-oxidants and coffee has anti-inflammatory effects, the physiologic basis for a purported effect is still unknown [49]. Small, short term (weeks) clinical trials have shown mainly negative results. The longest (16 weeks) trial, however, showed a slight, though questionable, benefit [50–53]. Moderate maternal consumption of caffeine during lactation does not appear to adversely affect the newborn, although more studies are needed [54].

Finally, advances in the use of internet and telephone-based interactions can facilitate interventions in the postpartum period, as they permit a more home-based approach. In at least one study these strategies have been found to be as effective as face-to-face coaching in achieving weight loss [55]. Additionally, barriers to successfully treatment of obesity in low resource settings may be overcome by the use of technology-assisted weight loss interventions. A recent systematic review has shown that compared to usual care, technology-assisted interventions in the primary care setting help patients achieve weight loss [56].

### Objectives

The aim of this study is to investigate the effectiveness of a lifestyle intervention program in delaying or preventing type 2 diabetes when implemented after a pregnancy complicated by GDM in women identified as being at higher risk.

Additionally, we aim to investigate factors related to success or failure in diabetes prevention, to develop materials and expertise to assist in the development of diabetes prevention programs, and to contribute to public policies for diabetes prevention in primary health care.

## Methods

### Design

**Fig. 1:**
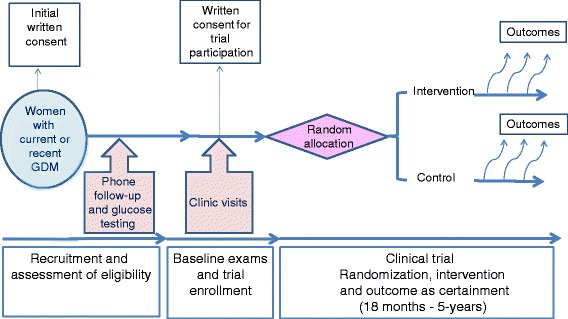
Description of the study protocol, including major actions taken during each of its phases

LINDA-Brasil is a multicenter randomized clinical trial to test the effectiveness of a lifestyle intervention program (of 18 to 60 months) in delaying or preventing type 2 diabetes when implemented shortly (10 weeks to 2 years) after a pregnancy complicated by GDM in women identified as being at higher risk (use of insulin during pregnancy and or post-partum lesser-than-diabetes hyperglycemia). Figure 1 illustrates the flow of participants through the trial, from recruitment to completion of the study.

### Recruitment and eligibility

All women aged 18 or older identified as having GDM at pre-natal clinics in selected Brazilian cities, regardless of the diagnostic criteria, and living within distance permitting an easy access to the trial sites, are invited to participate.

At first contact, the study protocol is explained and an initial signed consent is obtained. Information regarding socio-demographics, reproductive history, pre- and early gestational weight, gestational age, GDM diagnosis and treatment as well as dietary, physical activity and smoking habits is obtained through interviews and chart review. Folders regarding the importance of preventing diabetes and of breastfeeding are distributed. If recruited during pregnancy and the immediate postpartum period, women are monitored monthly by telephone until eligible to begin the trial. Date and place of birth are obtained and intention to breastfeed evaluated. Additionally, we inquire about the use of insulin and or oral antidiabetic medication during later pregnancy and obtain the last measured pregnancy and postpartum weights.

Although entrance is permitted up to 2 years postpartum, eligible women are encouraged to enter the trial as early as 10 weeks after pregnancy. Those who used insulin during pregnancy are automatically eligible. Eligibility for remaining women is assessed by a 2 h 75 g oral glucose tolerance test (OGTT) performed after the sixth postpartum week. Women presenting impaired fasting glucose (fasting plasma glucose ≥ 100 mg/dl; 5.6 mmol/L) or impaired glucose tolerance (2 h plasma glucose ≥ 140 mg/dl;7.8 mmol/L) [57] on this testing are eligible.

The following exclusion criteria apply:

- confirmed diabetes (two abnormal OGTTs), [57] or one OGTT with unequivocal values [abnormal fasting *and* 2 h plasma glucose values; fasting plasma glucose ≥140 mg/dl (7.8 mmol/L) or 2 h glucose ≥270 mg/dl (15 mol/L)];
- current use of antidiabetic medication;
- normal glucose tolerance (if not using insulin during pregnancy);
- an indication that the trial interventions are not feasible, for example, due to relocation to a place distant from the trial site or a poor response in previous contacts (e.g., lack of interest in phone interviews);
- a body mass index ≥ 40 kg/m^2^; and
- health limitations or treatments (assessed by questionnaire) which would restrict the nutritional intervention or the ability to practice physical activity, affect glucose tolerance or limit participation or survival.

With respect to this last criterion, the presence of the following chronic conditions indicate exclusion: cancer requiring treatment in the past 5 years (except those which have been cured or carry an excellent prognosis); certain infectious diseases (HIV positivity or active tuberculosis); clinically important cardiovascular disease (recent hospitalization or congestive heart failure requiring use of a diuretic or digitalis preparation); uncontrolled hypertension (systolic blood pressure ≥180 mmHg or diastolic blood pressure ≥105 mmHg on treatment); certain gastrointestinal diseases (chronic hepatitis or cirrhosis, alcoholic hepatitis or alcoholic pancreatitis; inflammatory bowel disease requiring treatment in the past year; recent or significant abdominal surgery (e.g. bariatric surgery); chronic renal failure; certain lung diseases (chronic obstructive pulmonary disease or asthma requiring daily therapy); a major psychiatric disorder (e.g. severe depression); and anemia not caused by iron deficiency.

Women not entering the trial are followed with yearly assessment of hyperglycemia until trial completion in order to derive external control estimates for the principal trial outcomes.

### Baseline measurements and trial enrollment

At baseline, women respond to a standardized questionnaire and undergo physical measurements. Data on prenatal care and delivery are extracted from the health care record carried by the participant. Questionnaires address risk factors for type 2 diabetes, including eating and drinking behaviors, physical activity [58], sedentary habits [59–61] as well as issues such as quality of life [62], depressive symptoms [63], perceived body image trajectories [64], quality and duration of sleep [65], medication use, and perceived risk of diabetes [66]. A physical examination includes blood pressure, waist and hip circumferences, weight, height, body composition, fitness assessment [67] and abdominal height [68]. Additionally, physical activity is assessed using accelerometers (ActiGraph GT3X) worn on the waist for seven days.

A standardized 75 g OGTT [57] is done with samples taken at fasting and 30, 60, 90 and 120 min [69]. A urine sample is collected upon arrival. Aliquots of serum, plasma, urine and DNA are stored for future measurements. To assure that women who used insulin during pregnancy and had not undergone a postpartum OGTT have not developed diabetes, their fasting and 2 h glucose values are determined prior to randomization. After key aspects of the clinical trial protocol are reviewed and remaining questions are clarified, an additional written informed consent is obtained and a further visit to the clinic is scheduled.

At this second baseline visit, women complete exams, including a six minute walking test. After eligibility has been once more confirmed (including the absence of diabetes), and key aspects of the protocol, including randomization, have been reviewed, women are enrolled into the trial.

Participants missing the scheduled visits are rescheduled when justified. Missing three appointments is a criterion for exclusion from the clinical trial. In this sense, these preparatory visits also serve as a run-in period for the trial.

### Randomization

Women are allocated to one of the two comparison groups. The computer generated randomization scheme is 1:1 by trial arm, stratified by center and performed in random blocks of sizes 4 and 6 participants. Treatment is allocated automatically, with clinical staff soliciting and then receiving the treatment allocation through the secure, password protected data entry system to guarantee adequate concealment of allocation.

### Intervention groups

- ✓ Control Group: Less intensive conventional care for women with prior GDM. This group receives a booklet containing instructions about diabetes prevention and materials based on current guidelines with recommendations for breastfeeding, physical activity and healthy eating. They are informed about the benefits of periodically checking their diabetes status and that such an assessment will be provided annually during the trial.
- ✓ Intervention Group: A more intensive program designed to promote and support healthy behaviors likely to prevent diabetes. In addition to receiving the above materials, women in this group receive participant-centered coaching focused on prolonging breastfeeding, weight control and a healthy life style with emphasis on the quality of their diet and physical activity. Coaching is primarily done by phone, and registration of weight and steps taken is encouraged.

### Key aspects of the intervention

Intervention is tailored so as to meet the particular needs of women with previous GDM during their postpartum. Building from social cognitive theory [70], interventions are participant-centered [71–73], allowing adaptation to each women’s setting, within the limits of a standardized prevention program with defined goals and structure. Communication strategies are based on motivational interviewing [14] and health coaching approaches implemented in the primary care setting [74].

The specific components of the intervention and corresponding goals are:

- Promotion and support for exclusive breastfeeding for up to six months of life and partial breastfeeding thereafter for at least three additional months.
- Stimulation and support for personally-monitored weight loss and maintenance with the goal of returning to pre-pregnancy weight and, for those overweight or obese pre-pregnancy, losing at least an additional 5 % of body weight. If this goal has already been achieved at randomization, the treatment goal will be a loss of 5 % of current body weight or that needed to achieve a BMI of 22.5 kg/m^2^.
- Promotion of healthy eating, emphasizing avoidance of ultra-processed foods, limited intake of oils, sugars and processed meats, increased intake of foodstuffs in their natural state, regular intake of water and dairy products, and intake of coffee and tea without sugar or sweeteners.
- Encouragement and support for a personally-monitored progressive increase in physical activity with an initial goal of breaking up extended bouts of sitting with ambulatory activity, reduction of sedentary behavior (defined as <5000 steps/day); and later striving to perform at least 150 min of moderate or vigorous physical activity/week (7500 steps/day with at least 30 min. taken at a cadence of 100 steps/min.) [75–77].

Table 1 summarizes the progressive nature of the lifestyle interventions which takes into consideration the particularities of recent motherhood, the gradual changes promoted, and the allowance of participant-centered approaches in the progression towards the final goals.

**Table 1:** Progressive nature of lifestyle interventions for weight control, healthy eating and an active lifestyle

	Stage 1	Stage 2	Stage 3
	8-12 weeks	8 a 26 weeks	Up to close out
Weight control	Initiating weight loss (up to 2 kg/month while breastfeeding) when needed.	Achieving further weight loss to goal when needed/weight maintenance.	Maintaining weight (additional weight loss if needed).
	Goal: return to pre-pregnancy weight and, for those overweight or obese prior to pregnancy, loss of at least an additional 5 % of body weight.		
Healthy eating	Developing healthy eating.	Overcoming difficulties to sustain healthy eating.	Maintaining healthy eating.
Active life style	Developing an active lifestyle.	Developing an active lifestyle.	Maintaining/enhancing an active lifestyle.
	Goal: breaking up extended bouts of sitting with ambulatory activity; reducing sedentary behavior (defined as <5000 steps/day).	Goal: increasing physical activity to at least 150 min of moderate or vigorous physical activity/week, or 7500 steps/day.	

During the first stage, coaching is conducted primarily to achieve gradual weight loss. Healthy eating and reduction of sedentary behavior are also emphasized and are the main focus for those who do not need to lose weight. Initial changes are promoted in accordance to participant desires and needs, and take into consideration the fact that most women will be breastfeeding (many exclusive breastfeeding) and still adapting to recent motherhood.

During the second stage, which is likely to occur after exclusive breastfeeding has terminated, while still aiming to achieve/maintain weight loss, we place emphasis on increasing physical activity. The third stage aims at maintaining progress achieved by monitoring goals, detecting relapses and preserving fidelity to the trial.

The main approaches used to deliver the intervention are described in Table 2, the principal approach being phone sessions, complemented by phone texting (SMS). Phone sessions occur initially at a weekly interval (3 sessions), then biweekly and, when weight goal is achieved, monthly for about one year. After the first year the frequency of phone sessions decreases and phone texting becomes the principal way of communication. Group sessions and social events are optional, to be used when felt needed throughout the study.

**Table 2:** Approaches of the intervention

Approach	Description
Motivational interviews	An initial 30–40 min session informs baseline results; evaluates motivation and self-efficacy; and sets individual goals to establish an initial action plan. Additional sessions occur during pre-defined clinic visits.
Phone sessions	Phone sessions are the principal means of delivering the intervention. A minimal core curriculum will be covered, but the sequence and frequency of the sessions will be tailored to each participant’s needs. During weight loss, frequency may be weekly or bi-weekly, but in general, monthly sessions are planned for the first year.
SMS texting	Texting is used for reminders of exams and monitoring of weight and steps. With the progression of the intervention, when phone contacts are reduced in frequency, texting will be used to maintain motivation and adherence.
Group sessions (as needed)	Specific topics are addressed, focusing on aspects which most benefit from group interaction. Participants are invited to attend sessions as needed.
Social activities (optional)	These optional activities include culinary workshops and group walks.

### Trial outcomes

Follow-up will continue until all women have completed at least 18 months of participation and the average length of follow up for the whole sample is at least three years. Data and safety monitoring issues are addressed in a specific section below. Strategies for maximizing adherence to the Study have been developed. Outcomes are assessed at baseline, then generally again at 6 months, and then annually. (Table 3)

**Table 3:** Measurements used to determine trial outcomes

Measurements	Times of measurement during follow up^a^	Main outcomes
Exams		
75 g oral glucose tolerance test	6 months, annual	Diabetes/intermediate hyperglycemia [57]; insulin resistance [69]
Glycated hemoglobin	6 months, annual	Diabetes [57]
HDL-C, triglycerides	6 months, annual	Metabolic syndrome [78]
Weight	6 months, annual	Weight loss/maintenance
Height		
Blood pressure, heart rate (Omron 765CP, Omron, Kyoto, Japan)	6 months, annual	Metabolic syndrome [78]
Waist and hip circumferences	6 months, annual	Metabolic syndrome [78]
Abdominal height (Holtain-Kahn caliper, Seritex, Tinton Falls, NJ, United States)	6 months, annual	
Percent body fat (InnerScan BC-1500,Tanita)	6 months, annual	
Handgrip strength (Jamar hydraulic hand dynamometer); 6 min walking test; sit and reach test (Wells bench, Wood-WCS)	6 months, annual	
Accelerometry (ActiGraph WGT3X)	6 months, annual	Reaching 7500 steps/day [75]
Questionnaires		
Quality of life [62]	Annual	
Depressive symptoms (Edinburgh questionnaire) [79]	6 months, annual	
Perceived body image [64] and diabetes risk [66]	Annual	
Sleep quality (Pittsburg questionnaire) [80]	Annual	
Breastfeeding status	6 months, 1 year	Duration of exclusive and overall breastfeeeding
Dietary recall/registry	6 months, annual	
Physical activity and sedentary behavior (IPAQ long version, leisure-time and transportation sections [58]; sedentary behaviors questionnaire) [59–61].	6 months, annual	

^a^All taken at baseline

Primary Outcome: The primary outcome is incident type 2 diabetes, which will be ascertained by OGTT and HbA1C in both groups. At study close-out, all women whose last ascertainment occurred more than 6 months previously will be reassessed.

Secondary Outcomes:

1. Normalization of intermediate hyperglycemia (fasting and 2 h blood samples, HbA1C) [57]
2. Metabolic syndrome (fasting blood samples, questionnaires) [78]
3. Mean insulin resistance, beta cell function (OGTT) [69]
4. Mean weight loss and weight goal achievement
5. Physical fitness (6 min. test [67], handgrip; sit and reach test)
6. Duration of breastfeeding and rate of exclusive breastfeeding up to 6 months postpartum (phone interviews between 6 months and 1 year postpartum)
7. Quality of life [62]
8. Mean body fat/central fat (weight, % body fat, waist circumference and abdominal height)
9. Sleep quality (Pittsburg questionnaire) [65]
10. Perceived body image (questionnaire) [64]
11. Depressive symptoms (Edinburgh questionnaire) [63]
12. Infant growth (reference curves)
13. Adverse events

Process outcomes:

1. Dietary quality (dietary recalls)
2. Intensity and duration of physical activity (Number of minutes spent in moderate to vigorous intensity activity and the number of minutes spent in sedentary activity, estimated by accelerometer; and percentage of women achieving the moderate or vigorous physical activity goal of 150 min/week, estimated by the leisure-time and transportation sections of the International Physical Activity Questionnaire - IPAQ, long version [58])
3. Percentage of women adhering to the protocol of intervention (phone sessions and outcome monitoring)
4. Participation in optional activities: group sessions and social events.

### Follow-up

The timeline of study activities during recruitment and the clinical trial is shown in Table 4. Follow-up of those not entering the trial is also included in the table.

**Table 4:** Timeline of main activities from recruitment to the end of the clinical trial

Activity	Recruitment for the trial and follow-up of those not entering the trial					Clinical trial						
	Pregnancy	Perinatal period	Post-pregnancy period (weeks)									
	Recruitment	Telephone contact	Trial preparation	Baseline measures	Follow-up^a^	All women entering the trial						
						Post-randomization follow-up (months)						
	Gestational weeks 20–40	Pregnancy to 30 days postpartum	4–104	8–104	Up to 260	0	0–6	6–12	12–24	24–36	36–48	48–60
Consents	x			x								
Entry	x	x	x			Trial randomization						
Interviews/exams/chart reviews	x			x		x		x	x	x	x	x
Phone contacts		x	x		x							
Baseline and follow-up measurements				x				x	x	x	x	x
Material distributions	Breastfeeding folder 1; Pregnancy to postpartum booklet	Mail at birth: Breastfeeding folder 2		Project folder		Preventing diabetes booklet						
						Intervention group						
Motivational interviews						x	x	x	x			
Phone sessions							x	x	x	x	x	x
SMS texting							x	x	x	x	x	x
Group sessions							As needed					
Social events							As needed					
Mailings							As needed					

^a^Follow-up of those not entering the trial

At trial follow-up visits, glucose determinations are performed by staff blinded to patient allocation. Principal investigators, clinical staff, outcome assessors and participants are blinded with respect to glucose results for the duration of the trial. Women who reach diabetes glucose levels are notified. Given that follow up the participants occurs at yearly intervals, we do not require confirmation of the diabetes status on another day to ascertain incident diabetes in order to avoid an excessive length of time prior to the initiation of medical care for diabetes.

### Sample size

We estimated that the control group will have a cumulative 3-year diabetes incidence rate of 25 %. This estimate lies between a lower rate (19 %) found in a trial involving women considerably leaner [9] and higher rates found in two trials: a trial of a very high risk postpartum group (36 %) [8] and another trial involving older women, enrolled a decade after their first pregnancy with GDM (38 %) [4]. We also assume that the intervention will produce a reduction of 40 % in diabetes incidence over three years. This estimated effect is lower than the incidence reduction observed in the DPP subgroup analysis (53 %), consistent with our anticipated intervention’s effect, and clinically meaningful for the prevention of diabetes in Brazil. The number needed to treat to prevent one case of diabetes is 10 (1/(.25-.15). Under an exponential model, the relative risk of 0.60 comparing intervention to control corresponds to a hazard ratio of 0.565.

We based our sample size calculation on a Cox proportional hazards model test of a one-sided primary hypothesis (no difference between treatments in incidence of type 2 diabetes) at the 0.025 level, with 90 % power, using the package RCTdesign in the software R, version 2.14. Utilizing a group sequential design, we plan for four analyses, three interim and one final with symmetric O’Brien-Fleming boundaries for efficacy and futility to be spaced evenly in information time. This analysis shows that 135 observed events in both arms are needed at the time of final analysis to have 90 % power to detect a true hazard ratio of 0.565. Assuming an annual drop-out rate of 5 %, an average monthly recruitment of 18 participants, and requiring an average follow-up time of three years if the study continues to the final analysis, we calculated a total sample size of 740 for the trial.

To enroll this number of women, we estimate that up to 7400 women will have to be initially screened. If we are unable to maintain our monthly recruitment goals with the strategy outlined above, we will consider other means of recruitment.

### Ethical issues

The trial protocol was approved by the ethics committee of the Hospital de Clínicas de Porto Alegre (Project 120097, May 4, 2012). Written consent is obtained at initial recruitment and then again, before enrollment to the trial. At randomization we explain to the participants that they will have equal chances of being entered into one or the other group. We also explain that it is not known whether applying a more intense intervention relatively soon after postpartum is more beneficial than the conventional approach. However, if the Study finds that it is indeed more beneficial, then at study close-out those who did not receive the more intense approach will be informed about how to undertake the activities found to be successful. While we emphasize the importance of maintaining participation throughout the study so as to minimize bias in the trial results, women are informed that they may request to terminate participation if they so decide. Those who drop out of the study will nevertheless have their available follow-up time included in analysis of treatment effects. Women found to have developed diabetes will be notified and oriented to seek medical attention to confirm diagnosis and initiate treatment.

A data and safety monitoring board (DSMB) will monitor efficacy (or futility) and adverse effects. Based on these considerations, the DSMB may recommend that the protocol be modified or that the LINDA trial be terminated. The DSMB will consist of experts in relevant biomedical fields, biostatistics and medical ethics.

### Final considerations

After testing instruments and piloting strategies and procedures, we initiated trial recruitment and randomization in January, 2015.

## Discussion

To our knowledge, this is one of the few randomized trials designed to prevent diabetes by intervening shortly after delivery of women with GDM. As such, the experience gained with respect to the specific issues related to intervention during this special phase of women’s lives should add important knowledge to the field of diabetes prevention worldwide.
